# Can 9q34.2 rs633862 polymorphism predict survival in epithelial ovarian cancer?

**DOI:** 10.7717/peerj.3946

**Published:** 2017-11-01

**Authors:** Rong Jiang, Yuan Xu, Pan Wang, Xi Cheng, Tingyan Shi, Rongyu Zang

**Affiliations:** 1 Ovarian Cancer Program, Division of Gynecologic Oncology, Department of Obstetrics and Gynecology, Zhongshan Hospital, Fudan University, Shanghai, China; 2 Cancer Institute, Fudan University Shanghai Cancer Center, Shanghai, China; 3 Gynecologic Oncology, Fudan University Shanghai Cancer Center, Shanghai, China

**Keywords:** *ABO* gene, Single nucleotide polymorphism, Epithelial ovarian cancer

## Abstract

**Objective:**

Our previous genome-wide association study (GWAS) identified that the * ABO* rs633862 variant in chromosome 9q34.2 was associated with the risk of epithelial ovarian cancer (EOC) in Chinese Han women. The aim of the present study was to evaluate its prognostic effect on EOC.

**Methods:**

A total of 669 EOC patients were enrolled for the genotyping of rs633862 variant in 9q34.2. We used Kaplan–Meier survival curves, univariate and multivariate Cox proportional hazard models to evaluate the association of rs633862 with overall survival (OS) in EOC patients.

**Results:**

We found that rs633862 variant AG/GG genotypes were significantly associated with a longer OS by using univariate Cox proportional hazards regression analysis, compared with the rs633862 AA genotype (HR = 0.69, 95% CI [0.49–0.98], *p* = 0.035), albeit with a boardline significance in the multivariate analysis. Similar findings were observed in the subgroup of high-grade serous ovarian carcinoma. Further expression quantitative trait loci (eQTL) analysis indicated that the rs633862 AA genotype was associated with an increased level of * ABO* mRNA expression (*p* = 1.8 × 10^−11^).

**Conclusions:**

Supplementary to the previous GWAS, our study provides additional evidence on the prognostic value of the 9q34.2 rs633862 variant in EOC patients, and this variant may function by regulating the * ABO* mRNA expression.

## Introduction

Ovarian cancer is a common gynecologic malignancy, the fifth leading cause of cancer death among women worldwide (Torre et al., 2015). In China, there were approximately 52,100 newly diagnosed cases and 22,500 deaths from ovarian cancer in 2015 (Chen et al., 2016). To date, the majority of epithelial ovarian cancer (EOC) patients are treated with primary debulking surgery followed by platinum/taxane chemotherapy, and show a good initial response. However, the five-year overall survival rate remains poor: nearly 30%–39% for advanced-stage disease (Pfisterer et al., 2006). Both clinicopathologic and genetic factors contribute to the mortality of EOC patients. In recent decades, genetic variants were found to play an important role in the development and progression of cancers, of which single nucleotide polymorphism (SNP) is the most common type that could influence patients’ prognosis (Li et al., 2013). Previous studies have identified several gene loci associated with clinical outcomes of EOC by using genetic association studies (Juan et al., 2016; Bolton et al., 2012), but the underlying mechanism remains unclear.

The *ABO* (ABO blood group) gene, located on chromosome 9q34, is well known to determine blood type. The encoded glycosyltransferases catalyze the transfer of nucleotide donor sugars to the H antigen and thus form the ABO blood group antigens (Yazer, 2005). Common variants lead to various glycosyltransferases and oligosaccharide antigens, and eventually cause four phenotypes: N-acetylgalactosamine for blood type A, D-galactose for blood type B, both for blood type AB, and neither for an unmodified H antigen in blood type O. Accumulated data have shown that the genetic diversity of the *ABO* loci may be caused by multiple alleles, various mutations and frequent recombination events (Hamosh et al., 2002; Yamamoto et al., 2012). The ABO blood group has been demonstrated to be associated with clinical outcomes of pancreatic cancer (Rahbari et al., 2012), esophageal squamous cell cancer (Sun et al., 2014), colon cancer (Cao et al., 2014), nasopharyngeal cancer (Ouyang et al., 2013), and breast cancer (Cihan, 2014). Recently, Cozzi et al. (2017) reported that blood type A was significantly associated with a longer survival in Caucasian ovarian cancer patients. However, in Chinese ethnics, the result was opposite (Li et al., 2015). In addition, to our knowledge, there was no report on the association between genetic variations in the *ABO* gene and ovarian cancer survival.

Our previous three-stage genome-wide association study (GWAS) has identified an EOC susceptibility-associated locus (rs633862) in the *ABO* gene on chromosome 9q34.2 (Chen et al., 2014). In 2015, a pooled analysis from the Ovarian Cancer Association Consortium (OCAC) and the Consortium of Investigators of Modifiers of *BRCA1/2* (CIMBA), also validated the role of 9q34.2 loci in a EOC risk estimate. Rs633862, on chromosome 9q34.2, is located at 5 kb upstream of the *ABO* gene, and has a minor A allele frequency of 0.456 in the Chinese Han population. However, its roles on ovarian cancer survival has not been well illustrated.

The present study aimed to identify the potential function of 9q34.2 rs633862 in EOC patients, hypothesizing that the rs633862 variant was associated with ovarian cancer survival. Based on the results of the previous three-stage GWAS (Chen et al., 2014), we here conducted a retrospective analysis among 669 Han Chinese patients with EOC.

## Materials and Methods

### Ethics statement

The study was approved by the Institutional Review Board of Fudan University Cancer Center (FUSCC) (Ethical Application Ref: 050432-4-1212B). A written informed consent was obtained from all recruited individuals.

### Study subjects

The patients were collected by the Shanghai Ovarian Cancer Study as described previously in the Chinese EOC GWAS, mainly from FUSCC between 2009 to 2012 (Shi et al., 2015). All patients were unrelated Han Chinese from eastern China, and histopathologically confirmed independently as primary EOC by gynecologic pathologists as routine diagnosis. As a result, a cohort of 699 patients with both GWAS genotyping data and available follow-up data were included into the analysis. The detailed clinical and pathological information of patients were extracted from the patients’ electronic database at FUSCC, included FIGO (International Federation of Gynecology and Obstetrics, 2013) stage, histopathology, tumor grade, pelvic lymph node (LN) metastasis, the expression of estrogen receptor (ER) and progesterone receptor (PR), chemotherapy schemes and residual disease after primary cytoreduction. For patients who received adjuvant chemotherapy with platinum and paclitaxel for six to eight cycles, follow-up was conducted in out-patient clinics or by telephone calls. Patients were followed every six months. If the patients could not be followed up for continuous three times, they will be considered as unavailable follow-up data and be excluded in the current study. Overall survival (OS) was defined as the time from the initiation of therapy (primary debulking surgery or first cycle of neoadjuvant chemotherapy) to the date of the last follow-up or the date to death of ovarian cancer. Patients without progression were censored at the date of last record.

### Genotyping

An additional 10 ml of venous blood sample (collected by the Tissue Bank of FUSCC after the diagnosis and before the initiation of treatment) were kept frozen till DNA extraction for genotyping. According to the standard protocol, genomic DNA was extracted from peripheral blood samples. Genotyping was performed using iPLEX MassARRAY platform (Sequenom, Inc., San Diego, CA, USA) as reported previously (Chen et al., 2014). Five percent of the samples were randomly selected for repeated genotyping, and the results were 100% concordant.

### Statistical analysis

We used Kaplan–Meier survival curves to visualize the OS by genotyping, and log-rank test for differences in the survival times among patients. Univariate and multivariate Cox proportional hazards models were used to evaluate the effects of genotypes and clinicopathological variables on patients’ OS by computing hazard ratios (HRs) and their 95% confidence intervals (CIs). Multivariate analyses were adjusted by those variables that were independently associated with survival in the univariate model. All of those tests were two-sided with a statistical significance set at *p* < 0.05, and were performed using SAS 9.1 software (SAS Institute, Cary, NC).

### In silico functional validation

We explored the function consequences of 9q34.2 (rs633862) using two *in silico* tools, including SNPinfo (https://snpinfo.niehs.nih.gov/) and the UCSC Genome Browser (http://genome.ucsc.edu) (Kuhn et al., 2007). To evaluate the association between rs633862 and gene expression in EOC, the publicly GTExPortal dataset (http://gtexportal.org) was used for the expression quantitative trait loci (e QTL) analysis.

## Results

### Associations between genotypes and overall survival

**Figure 1:**
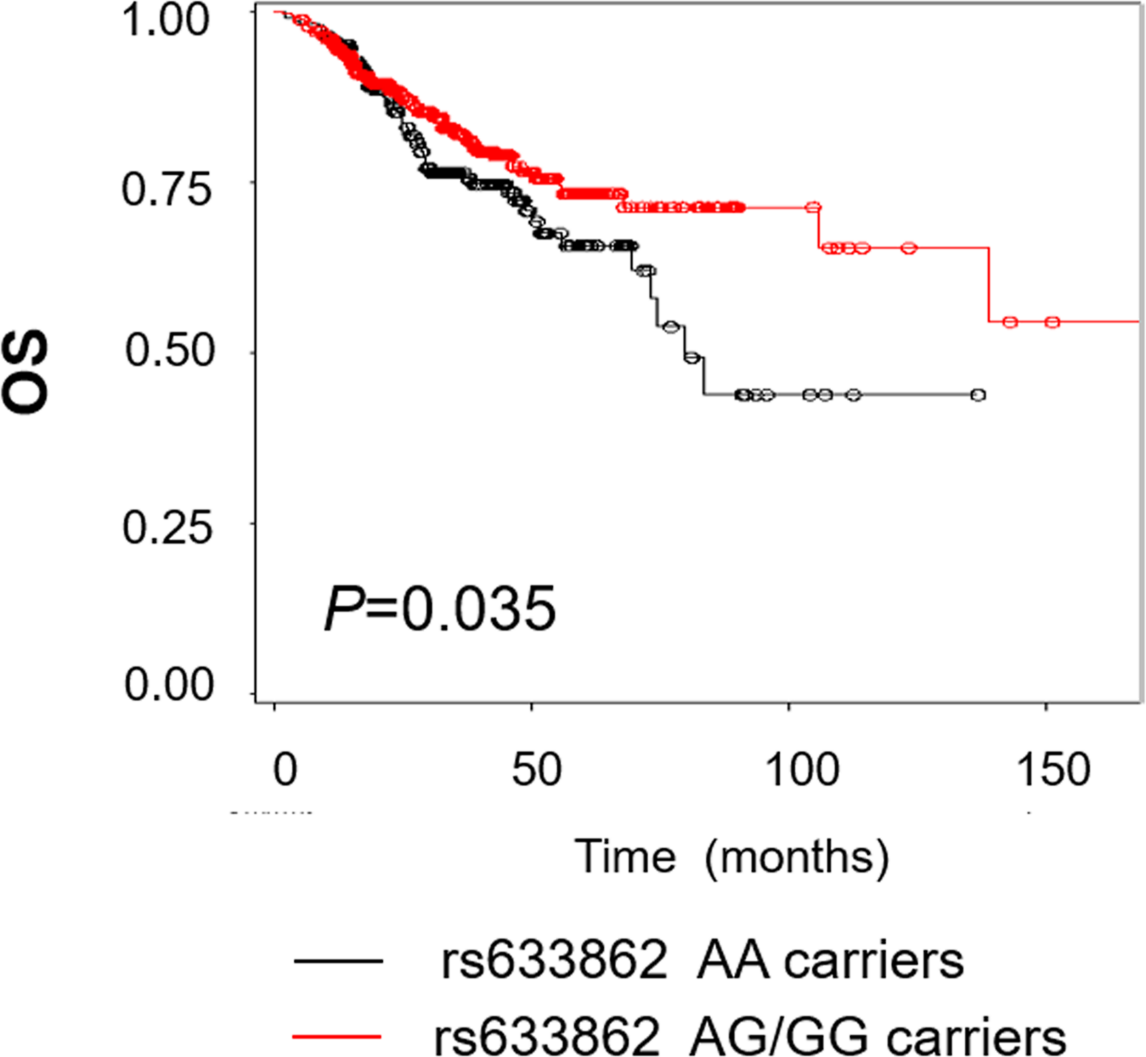
Kaplan–Meier estimates for the survival of patents according to rs633862 genoty pes. Rs633862 variant AG/GG genotype carriers was shown to have a significantly longer overall survival, compared with rs633862 AA genotype carriers (*HR* = 0.69, 95% CI [0.49–0.98], *p* = 0.035).

The detailed clinical and pathological information was listed in Table 1. Out of 669 EOC patients, 136 (20.3%) died at the last follow-up time (June, 2014), with a median overall survival of 31.5 months. As shown in Fig. 1, patients who carried rs633862 AG/GG genotypes had a longer OS, compared with AA genotype carriers (log-rank test, *p* = 0.035). Further univariate Cox proportional hazards regression analyses confirmed our data that rs633862 was significantly associated with EOC prognosis (AG/GG genotypes, *HR* = 0.69, 95% CI [0.49–0.98], *p* = 0.035), albeit with a boardline significance in multivariate analysis (*p* = 0.103) (Table 2). Interestingly, the similar prognostic value of rs633862 in overall survival was illustrated in the subgroup of high-grade serous ovarian carcinoma (univariate model: *HR* = 0.67, 95% CI [0.46–0.99], *p* = 0.046; Table 3). Moreover, age, FIGO staging, histological type and complete cytoreductive surgery were significantly associated with overall survival independently (*p* < 0.05 for all, Table 2). In the multivariate Cox proportional hazards model, age above 54.5 years old (adjusted *HR* = 1.04, 95% CI [1.01–1.06]), FIGO stage III–IV; (adjusted *HR* = 8.09, 95% CI [1.07–61.26]) and complete cytoreductive surgery (adjusted *HR* = 0.39, 95% CI [0.21–0.71]) remained statistically significant (Table 2).

**Table 1:** Clinical characteristics of epithelial ovarian cancer patients.

Characteristics	Patients	
	*N* = 669	%
Age, (mean ± SD), yrs	54.3 ± 0.39	
FIGO stage		
I	15	3.9
II	27	7.0
III	293	76.3
IV	49	12.8
Histopathology		
High-grade serous	476	71.3
Low-grade serous	76	11.4
Endometrioid	38	5.7
Clear cell	32	4.8
Mucinous	22	3.3
Others	23	3.5
Tumor grade		
Grade 1	11	1.9
Grade 2	100	16.9
Grade 3	481	81.3
Pelvic LN metastasis		
Negative	175	53.0
Positive	155	47.0
ER expression		
Negative	137	30.3
Positive	315	69.7
PR expression		
Negative	290	63.2
Positive	169	36.8
Residual disease after primary cytoreduction		
0 (no grossly visible tumor)	221	35.3
1 (0.1–0.5 cm)	113	18.0
2 (0.5–1.0 cm)	144	23.0
3 (>1.0 cm)	149	23.8
Recurrence		
No	173	25.9
Yes	496	74.1
Death		
No	553	79.7
Yes	136	20.3

**Notes.**

FIGO International Federation of Gynecology and Obstetrics LN lymph node ER estrogen receptor PR progesterone receptor

### Bioinformatics prediction between 9q34.2 rs633862 and ovarian cancer

**Figure 2:**
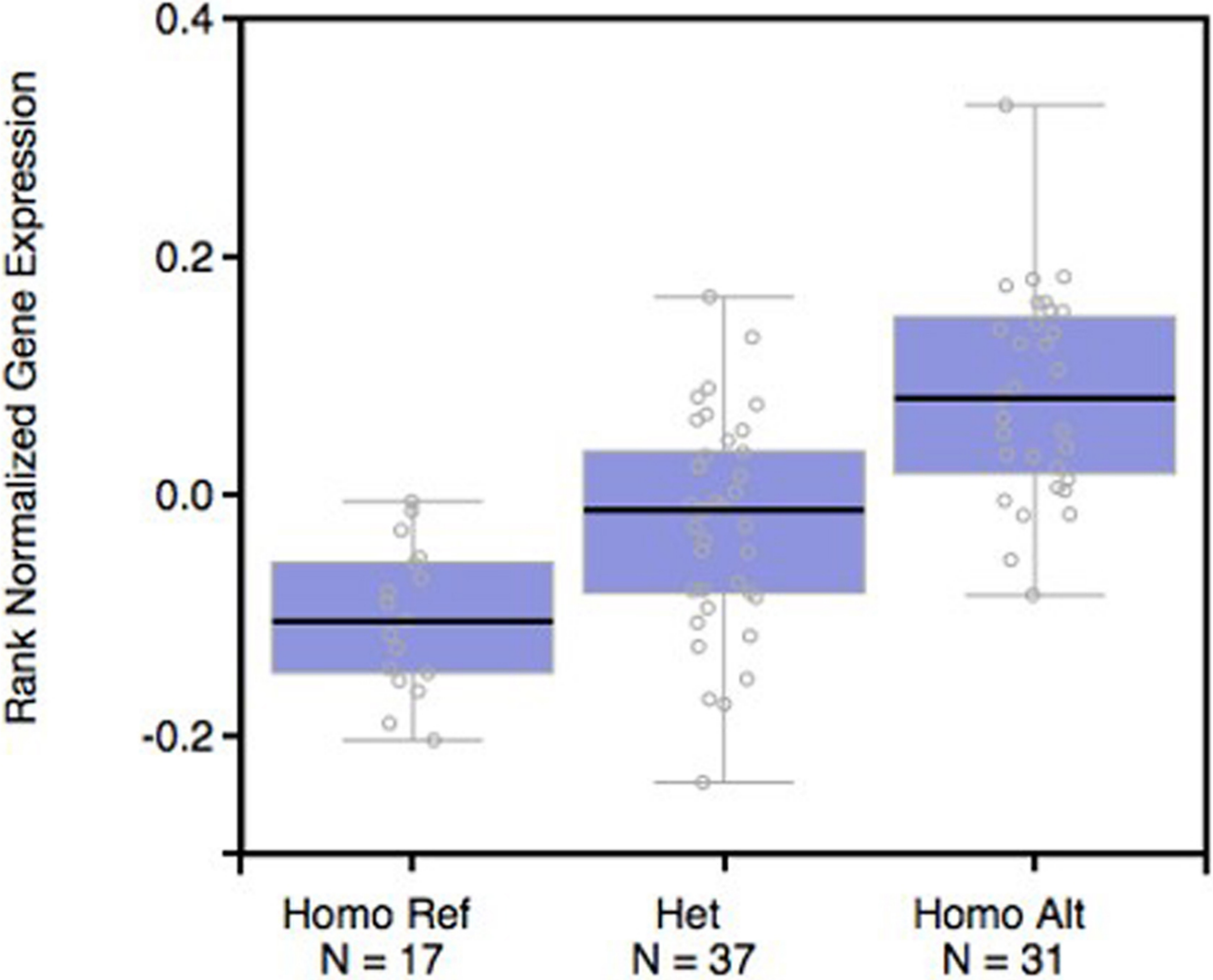
* ABO* mRNA expression by eQTL analysis in patients with EOC. The rs633862 AA genotype was associated with an increased level of * ABO* mRNA expression (*p* < 0.05). Homo Ref, rs633862 GG genotype; Het, rs633862 AG genotype; Homo Alt, rs633862 AA genotype.

We used the SNPinfo online tool to evaluate plausible biological mechanisms underlying the observed association, and found that 9q34.2 rs633862 was located at 4,814 bp upstream of the *ABO* gene, and predicted as a transcription factor binding site (TFBS) (Table S1). By using the public GTExPortal database, we evaluated the available *ABO* mRNA expression in relation to rs633862 genotypes in a total of 17GG, 37AG, and 31AA carriers. According to the eQTL information analysis (Fig. 2), rs633862 AA genotype was associated with an increased level of *ABO* mRNA expression (*p* = 1.8 × 10^−11^). These findings indicated that the rs633862 *G* → *A* variant may regulate *ABO* expression, leading to the increased mortality of EOC patients.

**Table 2:** Associations of variables with the survival of epithelial ovarian cancer by Univariate and Multivariate Cox proportional hazards models.

Variables	No. of cases	No. of death (%)	Univariate model		Multivariate model	
			HR (95% CI)	*P*	HR (95% CI)^a^	*P*^a^
**All**	669	136 (20.3)				
**Age, yrs**						
>54.5 (median)	332	77 (23.2)	1.61 (1.15–2.27)	**0.006**	1.04 (1.01–1.06)	**0.002**
**FIGO stage**						
III–IV	342	90 (26.3)	17.85 (2.48–128.76)	**0.004**	8.09 (1.07–61.26)	**0.043**
**Histopathology**						
HGSOC	476	103 (21.6)	1.60 (1.07–2.38)	**0.022**	1.12 (0.60–2.08)	0.715
**Complete cytoreduction**						
yes	221	19 (8.6)	0.22 (0.13–0.37)	<.**0001**	0.39 (0.21–0.71)	**0.002**
**Rs633862 genotype**						
AG/GG	438	77 (56.6)	0.69 (0.49–0.98)	**0.035**	0.70 (0.46–1.08)	0.103

**Notes.**

HGSOC high grade serous ovarian carcinoma

a Multivariate analyses were adjusted by those variables that were independently associated with survival in the univariate model; in this case, including age, FIGO stage, histopathology and complete cytoreduction.

**Table 3:** Prognostic value of rs633862 AG/GG genotypes in the subgroup of histopathology.

Variables	No. of cases	No. of death (%)	Univariate model		Multivariate model	
			HR (95% CI)	*P*	HR (95% CI)	*P*
**Histopathology**						
HGSOC	311	56 (18.0)	0.67 (0.46–0.99)	**0.046**	0.65 (0.41–1.03)	0.064
Non-HGSOC	126	21 (16.7)	0.80 (0.39–1.65)	0.550	1.01 (0.29–3.53)	0.990
Low-grade serous	53	11 (20.8)	0.69 (0.25–1.91)	0.476	0.32 (0.07–1.45)	0.138
Endometrioid	22	2 (9.1)	0.76 (0.11–5.37)	0.780	–	0.999
Clear cell	22	2 (9.1)	0.94 (0.09–10.39)	0.959	–	1.000
Mucinous	14	3 (21.4)	0.44 (0.09–2.21)	0.317	–	1.000

## Discussion

In this study, we investigated the associations between a potentially functional variant in chr9q34.2 (rs633862) and overall survival of EOC patients. Our data showed that patients carrying rs633862 AG/GG genotypes had a longer OS, compared with those with AA genotype. Furthermore, genotype-phenotype correlation analyses suggested that rs633862 involved in regulating *ABO* mRNA expression, indicating its potential function on modulating ovarian cancer progression.

Previous studies have demonstrated the association between ABO blood group antigens and clinical outcome in several human cancers, such as esophageal squamous cell cancer (Shiratori et al., 2017), laryngeal cancer (Jin et al., 2016), gastric cancer (Xu et al., 2016) (Angelov et al., 2014) and non-small cell lung cancer (Li et al., 2015). Also, the association between ABO blood group and ovarian cancer was reported, but the results were opposite between Caucasians and Chinese (Cozzi et al., 2017; Li et al., 2015). Moreover, the mechanisms of the ABO blood group on malignancies were still unclear. *ABO* gene encodes glycotransferases, which are a crucial catalyst in the formation of ABO antigens. The expression of the ABO antigen is not only on blood cell membranes, but also on the surface epithelium and inclusion cysts of the ovary (Welshinger et al., 1996). Blood group antigens expressed on tumor cells could influence tumorigenesis, cell motility and immune escape (Pendu et al., 2001). In addition, recent studies have reported that *ABO* types were associated with levels of soluble ICAM-1 (Paré et al., 2008), tumor necrosis factor-alpha (Melzer et al., 2008), and soluble E-selection (Qi et al., 2010), indicating the possible role of blood group antigens in immune response.

To the best of our knowledge, this is the first study demonstrating the predictive value of *ABO* variants in EOC among Han Chinese women. Interestingly, we found that the rs633862 AG/GG genotypes might be a protective factor for overall survival. Further bioinformatics analyses showed that the genomic region containing 9q34.2 (rs633862), located in the upstream of *ABO* gene, was predicted as a transcription factor binding site, and could affect the biosynthesis of protein. Genotype-phenotype correlation analysis from the online GTExPortal database indicated a significant association of *ABO* mRNA expression levels with rs633862 genotypes. Therefore, we concluded that rs633862 AA genotype may function by up-regulating *ABO* mRNA expression and thus contribute to a worse OS in Chinese EOC patients.

Several limitations in the present retrospective study need to be addressed. First, the sample size and the follow-up time seems relatively limited, which might be a main reason for the boardline significance of rs633862 in the multivariate model analysis. Second, we used bioinformatics to predict the potential function of *ABO* gene, further validation of genotype-phenotype correlation by using the current study samples and functional experiments focused on rs633862 variant are needed to illustrate the potential mechanisms. Third, further studies should include more polymorphisms in the *ABO* gene and related regions.

In summary, as the supplementary of the previous GWAS, our study provides an additional evidence of 9q34.2 rs633862 variant on ovarian cancer prognosis. Rs633862 AA genotype may function by up-regulating *ABO* mRNA expression, and thus contribute to clinical outcomes in Chinese ovarian cancer patients. However, well-designed larger, prospective studies with longer follow-up time and further functional analyses are warranted to validate our findings.

##  Supplemental Information

10.7717/peerj.3946/supp-1 Table S1

10.7717/peerj.3946/supp-2 Data S1 Raw data

## Additional Information and Declarations

### Data Availability

The following information was supplied regarding data availability: The raw data has been uploaded as a Supplemental File.
